# Intrapersonal and Interpersonal Concomitants of Facial Blushing during Everyday Social Encounters

**DOI:** 10.1371/journal.pone.0118243

**Published:** 2015-02-13

**Authors:** Marije aan het Rot, D. S. Moskowitz, Peter J. de Jong

**Affiliations:** 1 Experimental Psychopathology and Psychotherapy Program, Department of Psychology, University of Groningen, Groningen, Netherlands; 2 Department of Psychology, McGill University, Montreal, Canada; Utrecht University, NETHERLANDS

## Abstract

Facial blushing may usually be undesirable but may have an ameliorative function for some individuals under some circumstances. Researchers have studied the blush in laboratory settings, but not in daily life. In the present research, conducted with young adults, we employed for the first time an event-contingent recording method for assessing facial blushing during every-day social encounters. Blushing was associated with feeling embarrassed, ashamed, and exposed. These findings, though based on correlational analyses, are consistent with the idea that blushing is often unpleasant and can be maladaptive, and may contribute to the common belief that blushing is an undesirable response. Frequent blushers generally reported lower levels of dominant behavior, higher levels of submissive behavior, and perceived their social interaction partners as more powerful and less affiliative. This was independent of whether they blushed or not, suggesting that altered social behaviors and perceptions are associated with blushing-associated traits rather than with the blushing state. The experience of the blush varied as a function of the frequency with which a person blushed. Blushing was associated with higher levels of shame in frequent blushers than in infrequent blushers. In infrequent blushers, blushing was associated with higher levels of pleasant affect, suggesting that for infrequent blushers the blush may occur in positive social encounters.

## Introduction

There have been conflicting theories and results concerning whether blushing is associated with pleasant or unpleasant experiences and whether the blush has communicative functions. In *The Expression of the Emotions in Man and Animals*, Darwin devoted a chapter to blushing but downplayed its interpersonal functions [1]. Recently, Crozier and de Jong argued that blushing is often associated with embarrassment and shame, intrapersonal emotions that cause distress, but they also presented evidence supporting the signal value of displaying a blush in social interaction [2].

Past research on blushing has mostly been limited to questionnaire and laboratory studies. Our goals were to study the intrapersonal and interpersonal concomitants of the blush naturalistically and to explore between-person differences in the within-person associations of blushing with emotions, behaviors, and perceptions. While this approach is correlational, it may constitute an important step in uncovering which factors are likely to elicit a blush in daily life, and how blushing during every-day social interactions might influence the outcome of these interactions.

### Blushing may be maladaptive for some people

Blushing refers to the sudden, involuntary reddening of a person’s face in situations in which the person is unwillingly the center of attention [3]. Several studies have suggested that blushing can be maladaptive. Using a large student sample, Bögels and colleagues found a positive correlation between social anxiety and retrospectively reported frequency of blushing [4]. Moreover, subjective blushing propensity has been linked to a heightened fear of negative evaluation [5]. The act of blushing may be both a source and a consequence of anxiety.

Blushing is not only associated with social anxiety, but also with the tendency to experience embarrassment during interactions with others [5]. Blushing often occurs in situations experienced as embarrassing by the blusher [6–9] and perceived as embarrassing by the observer of the blush [10]. Similarly, blushing often occurs in situations experienced or perceived as shameful [10]. Crozier has used literary examples of blushing to argue that blushing in situations in which someone feels embarrassed or ashamed is best explained by a general sensitivity to evaluation by others, which could be negative, positive, or neutral in tone, or by the anticipated exposure of something personal that the blusher prefers to keep private [11,12].

Blushing may also occur in the absence of embarrassment, shame, or exposure. Feeling self-conscious may be sufficient, since people often blush in situations in which they care about their appearance to others [11,12]. As Darwin observed [1], blushing can be linked to shyness, a trait that reflects a tendency to feel exposed and self-conscious regardless of the situation [13]. Shy people are cautious to prevent social norm violations (mishaps) and ethical or moral transgressions, as these situations would lead them to feel embarrassed or ashamed. Shy people are also likely to show signs of submission and affiliation even in neutral situations. Thus, shyness may be seen as a proactive form of appeasement. However, as they fear evaluation, shy people may also blush in situations in which most people do not, thereby ironically generating cause for embarrassment and shame.

Blushing may be an important source of fear in individuals diagnosed with social anxiety disorder. These individuals are considered pathologically shy [14]. They avoid being the center of attention, engaging in situations in which something personal might be exposed, and having social interactions during which they might otherwise feel self-conscious [15]. Individuals with social anxiety disorder report that they blush frequently [16]. If they are very fearful of blushing, then they often have strong dysfunctional beliefs about blushing [15,17]. These beliefs make individuals with social anxiety disorder relatively likely to blush [18], which reveals their shyness. Thus, there are traits which characterize individuals for whom blushing is particularly likely to be associated with maladaptive consequences.

### Blushing may be adaptive in some situations

People do not always blush when they feel embarrassed or ashamed during interactions with others, but blushing during interactions in which embarrassment or shame occurs may yield a more positive outcome. This includes blushing in the context of social mishaps such as spilling coffee on someone, and even blushing in the context of more serious transgressions such as stealing [19,20].

Following a mishap, people tend to think that others form an undesirable impression of them, and this is associated with embarrassment [21]. Humans express embarrassment by averting the eyes, turning the head away, suppressing the smile, and touching the face [22,23]. These expressions serve to reduce aggression, to appease, and to enhance bonding in others, thereby bringing about reconciliation and repair of the relationship. A person who expresses embarrassment after a mishap is often assigned more positive character traits and liked more, and the mishap is more often perceived as amusing, compared to when the person does not express embarrassment [13]. Notably, these effects on others’ perceptions are enhanced when the person blushes [19]. Thus, blushing after a mishap may help avoid conflict and maintain interpersonal bonds.

The effects of becoming the center of attention after a mishap are often not negative, probably because mishaps tend to happen unintentionally. In contrast, when people become the center of attention after an ethical or moral transgression, others may perceive this as a character flaw, leading to the experience of shame [24]. In the presence of others, shame is usually expressed by a lowered eye gaze, a downward head tilt, and a slumped posture [23,25]. A person experiencing shame may blush, which reduces the chance that others will perceive the transgression as intentional, serious, and offensive [20]. Blushing also promotes trust behavior in others even after they have been betrayed [26]. Thus, blushing restores trustworthiness and can help “save face” even after a serious transgression.

The blush involves an acute accumulation of blood in the superficial venous plexus of the skin [27,28]. It reflects a neural vasodilator response that is controlled by the sympathetic branch of the autonomic nervous system [29]. Consequently, it is not possible to intentionally elicit a blush when it would be helpful to do so, nor to inhibit the blush when this would be helpful. Blushing may be adaptive and restore relationships, because it is never voluntary and must therefore be a genuine sign of embarrassment or shame.

### Limitations of past studies on blushing

Research thus far has primarily focused on the negative aspects of blushing [4,5]. While theories on the adaptive functions of the blush have also been developed [10,19,20,26,30], the strength of the association between blushing and negative versus positive outcomes remains unclear. The impact of blushing may vary with the social context such as the number of perceivers present [28,31] and whether the blusher is uncomfortable with these perceivers [31]. Moreover, the impact of blushing may vary with stable characteristics of the blusher such as trait anxiety and blushing frequency.

To date, the intrapersonal and interpersonal concomitants of the blush have primarily been examined in studies conducted in laboratory settings. Several of these studies used vignettes to assess intra-individual variation in the social contexts that may elicit blushing, and in self-reported responses to blushing by others [19,20]. Studies on individual differences in blushing propensity and fear of blushing have used trait measures administered at single time points [4,15,20]. These studies were limited in that participants were asked to imagine themselves in hypothetical situations or to recall past events. People are not always able to report how they would respond in real life [32] and their episodic memory is often poor, particularly if they are socially anxious [33]. With a few exceptions [18,28], laboratory studies have mostly focused on emotions and thoughts in relation to imagined blushing, presumably because it is extremely difficult to bring blushing under experimental control.

### The present research

To increase insight into the social correlates of the blush we moved from the laboratory to daily life. We sampled social encounters repeatedly and intensively in naturalistic settings, using an event-contingent data recording approach. In event-contingent recording (ECR) studies, to minimize retrospection, participants are asked to report on certain events of interest soon after they occur [34]. For blushing, social encounters are the relevant events.

We collected ECR data in two samples of first-year university students. Individuals in this age group blush often [31] and are relatively likely to be diagnosed with social anxiety disorder [35]. The procedures for Sample 1 and Sample 2 were identical except in Sample 2 we also collected questionnaire data on blushing-associated traits. Samples 1 and 2 were recruited separately for two reasons. First, results obtained from Sample 1 were considered preliminary data that required replication. Second, by asking Sample 2 to complete blushing-associated trait measures, we were able to test several additional hypotheses.

Initially we set out to examine how often people blush and in which social contexts. Using ECR we could assess blushing frequency in real-time, while previous studies have employed retrospective measures. Bögels and colleagues found a self-reported mean of 2.7 blushing events per week in a large sample of university students [4]. Shields and colleagues asked men and women of different ages to estimate their blushing frequency [31]. Some individuals reported blushing daily, while others reported blushing less than once a month. Thus, we hypothesized that most participants would report blushing at least once during a period of two weeks, and that there would be a substantial range in blushing frequency. Consistent with previous laboratory studies [6,28], we also hypothesized that women would blush more often than men and that blushing would be relatively common in group settings.

After this initial step, we focused on the intrapersonal and interpersonal concomitants of the blush. With respect to how people feel during their interactions with others, previous ECR studies have mostly focused on pleasant and unpleasant affect [34]. As feeling embarrassed, ashamed, exposed, and self-conscious is often linked to blushing, we also asked participants to what extent they experienced these feelings [1,4,11,12,15,31]. In line with previous research [1,4,11,12,15,31], we hypothesized that participants would report higher levels of embarrassment, shame, self-exposure, and self-consciousness when they blushed than when they did not blush.

Quarrelsomeness-agreeableness and dominance-submissiveness represent a person’s need for affiliation and his or her need for status or power, respectively. Affiliation and power are labels often given to the two orthogonal dimensions that define the interpersonal circumplex model [36]. Perceiving quarrelsomeness in others often evokes quarrelsome behavior and perceiving agreeableness often evokes agreeable behavior, while perceiving dominance in others often evokes submissive behavior and perceiving submissiveness often evokes dominant behavior [37,38]. The ECR approach we used [39,40] allows for the simultaneous characterization of a person’s social behaviors and his or her perceptions of others in terms of quarrelsomeness-agreeableness and dominance-submissiveness. Thus, we examined the interpersonal concomitants of the blush within the context of the interpersonal circumplex model. Since people who are feeling embarrassed or ashamed use motor displays of submission and affiliation [13,23], we hypothesized that blushing would be associated with high levels of submissive and agreeable behavior. Furthermore, consistent with research indicating that people blush more when interacting with people of a higher status [31], and with the principles of complementarity which predict that people behave submissively and agreeably when they perceive their interaction partner as dominant and disagreeable [37,38], we hypothesized that blushing would be relatively likely to occur during interactions with others perceived as powerful and low in affiliation.

Our primary research question was, how do people feel, behave, and perceive others when they blush compared to when they do not blush? As the social correlates of the blush may differ between frequent and infrequent blushers [41–43], our secondary research question was whether people who blush frequently feel, behave, and perceive others differently during blushing and non-blushing events than people who blush infrequently. Individuals with social anxiety disorder report that they blush often [16], engage in more submissive behaviors than controls [44], and perceive others as less affiliative [45]. Moreover, Russell and colleagues found that when individuals with social anxiety disorder feel anxious (and thus may be relatively likely to blush) their levels of submissive behaviors are particularly high [44]. Thus, we hypothesized that the association between blushing and submissive behavior would be stronger for frequent blushers than for infrequent blushers. Conversely, because blushing might be more successful at repairing relationships for infrequent blushers than frequent blushers, we hypothesized that people who blush infrequently would perceive others as more affiliative during blushing events than frequent blushers.

## Methods

### Ethics Statement

We obtained approval to conduct the study from the Ethics Board of the Department of Psychology at the University of Groningen and obtained written informed consent from all participants as described under Procedures. We conducted the study in accordance with the Declaration of Helsinki.

### Participants

First-year students in the English-language Bachelor program in Psychology at the University of Groningen participated in the study for educational purposes. We used a website exclusively accessible to first-year Psychology students to recruit participants for a study on every-day social functioning. There were no selection criteria. Students received partial course credit for their participation.

Samples 1 and 2 were recruited in successive years to provide a sample large enough to have sufficient statistical power for testing the moderating influences of individual differences in blushing frequency on how people feel, behave, and perceive others when they blush compared to when they do not blush. Two Sample 1 recruits and ten Sample 2 recruits were excluded from the data analyses because they did not return the ECR forms as per instructions or attend the second laboratory visit. Sample 1 participants who completed the study (*n* = 64, 55% female) ranged from 18 to 27 years of age, *M* = 20.46, *SD* = 1.53. Sample 2 participants who completed the study (*n* = 64, 42% female) ranged from 18 to 34 years of age, *M* = 21.63, *SD* = 3.05. The average age was 21.05 years across all participants. Men, *n* = 68, *M* = 21.65 years, *SD* = 2.86, were older than women, *n* = 62, *M* = 20.40 years, *SD* = 1.79, *t*(110) = 2.98, *p* < 0.004. Only 6% of participants were native speakers of English, but before admittance to the Bachelor program all were tested on being proficient to nearly-proficient users of English.

### Event-contingent recording

To sample social encounters from every-day life, we used the event-contingent recording (ECR) method developed by Moskowitz [39]. ECR represents a form of experience sampling or ecological momentary assessment that focuses on how people behave during their interactions with others [34]. A social encounter was defined as a spoken conversation, in person or on the phone, lasting at least five minutes. For the 14 days of the study, participants were instructed to complete standardized forms soon after an encounter had occurred. Each form included a list of unpleasant and pleasant affect items [46]. Participants used a scale from zero (“not at all”) to six (“extremely”) to indicate how they felt during a social encounter. To create an unpleasant affect score, ratings on the unpleasant affect items (worried/anxious, frustrated, angry/hostile, unhappy, and depressed/blue) were first summed and then divided by five, which was the number of negative adjectives. To create a pleasant affect score, ratings on the pleasant affect items (happy, pleased, joyful, and enjoyment/fun) were first summed and then divided by four, which was the number of positive adjectives.

Each form included three quarrelsome (e.g., “I made a sarcastic comment”), agreeable (e.g., “I exchanged pleasantries”), dominant (e.g., “I made a suggestion”), and submissive (e.g., “I spoke softly”) behaviors. Participants were asked to check the behaviors they engaged in during each social encounter. The Social Behavior Inventory provides 12 items for each of these behavioral dimensions and has been shown to provide valid and reliable scores for these dimensions [34,39]. The list of behaviors on a form varied in a daily rotation of four forms to prevent participants from developing a response set. Scores for each of the four behaviors were calculated for each record form by computing their individual mean frequency and then subtracting the mean frequency for all behaviors. These ipsatized scores reflect the frequency with which the different behavior types were checked, adjusted for the general rate of behavior checking.

Each form also included an interpersonal grid [40]. Participants indicated the extent to which they perceived their social interaction partner as affiliative (i.e., agreeable versus quarrelsome) and powerful (i.e., dominant versus submissive). Participants were instructed to complete the 11 by 11 grid with a single mark when interacting with one other person and when interacting with a primary other in a group, but to leave it blank when no primary other could be identified. Higher scores on the horizontal axis indicated that others were perceived as more affiliative. Higher scores on the vertical axis indicated that others were perceived as more powerful.

The forms as described so far have been used in previous ECR studies. For the study of blushing we adapted the forms in two ways. First, to the list of social behaviors we added an item on blushing: “I started blushing” (form 1), “I turned red” (form 2), “I suddenly became hot in the face” (form 3), and “I became flushed” (form 4). Second, to the list of affect items we added embarrassed, ashamed, exposed, and self-conscious.

### Procedures

Individuals who were interested in the study based on the advertisement used an online system to sign up for an individual meeting with a research assistant in a university laboratory. Upon arrival, they read a study information sheet and discussed it with a research assistant. The study rationale was explained in terms of obtaining data on social encounters in real-time rather than by using retrospective measures; there was no information about blushing. After providing written informed consent, both samples completed several retrospective measures. Sample 1 completed the Quick Inventory of Depressive Symptoms, Self-Report version (QIDS-SR) [47] and the Revised Leiden Index of Depression Sensitivity (LEIDS-R) [48]. These measures are not considered in the present study. Sample 2 completed the Social subscale of the Fear Questionnaire (FQ-Social) [49], the Blushing subscale of the Fear of Blushing, Trembling, and Sweating Questionnaire (BTSQ-Blushing) [15], and the Brief Fear of Negative Evaluation scale (BFNE) [50]. The FQ-Social was used to assess the tendency to avoid socially evaluative situations, the BTSQ-Blushing was used to assess fear of blushing, and the BFNE was used to assess fear of socially evaluative situations.

All participants received detailed instructions on the ECR approach. They were instructed to complete as many ECR forms as possible, up to 10 per day, and to disperse completion throughout the day. They were given packages containing 14 envelopes, each with 10 record forms. All of a day’s forms were to be returned as soon as possible after completion. To facilitate return of the forms, an envelope collection box was positioned at the entrance of the departmental building. Envelopes were labeled with the participant number and the study day. Sample 1 participants completed a mean number of 13.48 days of ECR (*SD* = 1.01) and returned a mean number of 63.13 forms (*SD* = 30.06). Similarly, Sample 2 participants completed a mean number of 13.31 days (*SD* = 0.91) and returned a mean number of 68.58 forms (*SD* = 30.49). On average, participants in the combined sample completed 13.56 days of ECR and returned 65.85 forms. Women, *M* = 76.63, *SD* = 30.78, returned more forms than men, *M* = 55.73, *SD* = 26.23, *t*(126) = 4.14, *p* < 0.0001.

Participants returned to the laboratory within a week after ending the ECR period. Sample 2 (but not Sample 1) completed the Blushing Propensity Scale (BPS) [5] and the Social Interaction Anxiety Scale (SIAS) [51]. The BPS was used to assess the self-reported tendency to blush often and the SIAS was used to assess fear of social interactions. All participants completed a feedback questionnaire about their experience with the record forms.

### Data analyses

Our data is freely available upon request. We removed all events that took place by phone (Sample 1: 14.45%; Sample 2: 8.30%) and all forms completed within three hours of alcohol ingestion (Sample 1: 6.50%; Sample 2: 7.34%). To test our hypotheses, the remaining ECR data (Sample 1: 3250 events; Sample 2: 3791 events) were analyzed with multilevel models and maximum likelihood estimation using PROC MIXED (SAS version 9.3, SAS Institute: Cary, NC). The degrees of freedom for *F* tests were determined according to Kenward and Roger [52]. All models included a random intercept and the default error covariance matrix. Analyses included Blushing, a dichotomous event-level variable (Yes, No) nested within participants; Blushing Frequency, a continuous participant-level variable based on the percentage of events that involved blushing; and the two-way interaction. The two-way interaction was tested because we expected the associations between (state) blushing and other variables (e.g. feelings experienced during social encounters) to differ between people with a higher (trait) blushing frequency and those with a lower (trait) blushing frequency. Blushing frequencies were determined by how often participants reported blushing during their social encounters. Significant interaction terms were examined by estimating simple intercepts and slopes for Blushing Frequency scores that were 1 SD above the sample mean (“frequent blushers”) or 1 SD below (“infrequent blushers”), and testing the significance of the difference between two slope estimates [53]. The significance level was set at 0.05.

## Results

### Internal consistency of the blushing items

We calculated mean scores on the four blushing items per participant. The Cronbach coefficient α in the combined sample was 0.65, indicating moderate reliability across different days and a wide variety of social encounters.

The item “I started blushing” had the lowest correlation with the total (*r* = 0.34), followed by “I suddenly became hot in the face” (*r* = 0.41), “I became flushed” (*r* = 0.46), and “I turned red” (*r* = 0.52).

### Stability of blushing frequency

The mean blushing frequency in the combined sample was 7.89% (*SD* = 8.29) and the median was 5.41% (range 0–45%). The mean blushing frequency was similar in the two subsamples (Sample 1: 7.89%; Sample 2: 7.88%).

We examined whether blushing frequency was a stable characteristic in the combined sample by correlating the frequency of blushing during week 1 with the frequency of blushing during week 2. Stability was found to be moderate, *r*(128) = 0.49, *p* < 0.0001.

### Gender differences

Gender differences were found in both samples. Women in Sample 1 were more likely to report blushing than men; women: *M* = 9.28%, *SD* = 7.61, men; *M* = 6.21%, *SD* = 7.48, *z* = 1.91, *p* < 0.03. Similarly, women in Sample 2 were more likely to report blushing than men; women: *M* = 9.79%, men: *M* = 6.49%, *SD* = 9.27, *z* = 2.14, *p* < 0.02. Sample 1 included 4 women and 10 men who never blushed. Sample 2 included 4 women and 14 men who never blushed.

### Social contexts of blushing

Blushing frequencies in different social contexts were compared using Wilcoxon signed-rank tests for repeated measurements of skewed data. We considered the following contextual factors: group size, partner sex, and partner role. Each analysis included only participants reporting social encounters in both contrasted contexts (e.g. with same-sex and opposite-sex partners).

Blushing frequencies were marginally higher during interactions with multiple others, *M* = 9.05%, *SD* = 11.14, than during interactions with one other, *M* = 6.95%, *SD* = 9.14, *S* = 511.5, *p* < 0.07, *N* = 127. Blushing frequencies were significantly higher during opposite-sex interactions, *M* = 9.57%, *SD* = 12.97, than during same-sex interactions, *M* = 5.91%, *SD* = 9.57, *S* = 632.5, *p* < 0.002, *N* = 124; and during interactions with romantic partners, *M* = 11.31, *SD* = 21.58, than during interactions with friends, *M* = 5.46, *SD* = 11.28, *S* = 98.5, *p* < 0.04, *N* = 55. Blushing frequencies were not significantly higher during interactions with higher-status others, *M* = 6.90, *SD* = 19.31, than during interactions with same-status others, *M* = 5.91, *SD* = 11.65, *S* = 6, *p* > 0.84, *N* = 56.

Since blushing frequencies varied with social context, in the subsequent analyses we statistically controlled for whether social encounters occurred in a group, for location, and for both participant gender and partner gender.

### State and trait: Blushing events in more and less frequent blushers

We examined Blushing (a state variable), Blushing Frequency (a trait variable, log-transformed and standardized to a mean of zero and a standard deviation of one), and the Blushing by Blushing interaction as predictors of emotions, behaviors, and perceptions. These analyses were conducted in the combined sample, see Table 1 for the *F*-statistics. Adding Sample as a moderator to the analyses did not meaningfully change the results; the Blushing by Sample interaction, the Blushing Frequency by Sample interaction, and the Blushing by Blushing Frequency by Sample interaction were not significant (data not shown).

**Table 1:** State-trait analyses for the combined sample.

	Blushing frequency (trait)	Blushing (state)	Blushing frequency by Blushing (state-trait interaction)
***Emotions***			
Embarrassment	27.77***	138.52***	8.37**
Shame	37.44***	48.32***	13.18***
Exposure	17.43***	60.07***	2.61
Self-consciousness	0.49	15.33***	4.69*
Unpleasant affect	22.60***	9.31**	3.30
Pleasant affect	0.43	4.90*	4.50*
***Social behaviors***			
Dominance	5.72*	0.12	1.69
Submissiveness	6.54*	0.03	1.50
Quarrelsomeness	2.17	0.64	2.30
Agreeableness	2.10	0.25	1.46
***Participants’ perceptions of others***			
Power	4.17*	0.13	0.08
Affiliation	4.38*	0.50	0.05

Note: *p < 0.05, **p < 0.01, ***p < 0.001. Values in cells represent *F*-statistics. Blushing frequency was log-transformed prior to the analyses.

**Blushing-associated emotions.** Embarrassment and shame were significantly predicted by Blushing, Blushing Frequency, and the interaction term. The feeling of exposure was significantly predicted by Blushing and Blushing Frequency. Self-consciousness was significantly predicted by Blushing Frequency and the Blushing by Blushing Frequency interaction (see Table 1). As levels of embarrassment, shame, exposure, and self-consciousness during social encounters that involved blushing were inter-correlated (all *r*’s > 0.16, all *p*’s < 0.0002), we repeated the analyses for each emotion while controlling for the other emotions. We subsequently describe those results.

Embarrassment was significantly predicted by Blushing, *b* = 0.47, *F*(1,6987) = 73.64, *p* < 0.0001, *d* = 0.21, and Blushing Frequency, *b* = 0.14, *F*(1,470) = 6.97, *p* < 0.009, *d* = 0.24. The interaction term was not a significant predictor, *F*(1,7023) = 1.82, *p* > 0.17. Participants reported more embarrassment when they blushed, *M* = 0.56, *SE* = 0.13, than when they did not blush, *M* = 0.09, *SE* = 0.11. Frequent blushers reported more embarrassment, *M* = 0.42, *SE* = 0.12, than infrequent blushers, *M* = 0.22, *SE* = 0.13.

**Fig 1:**
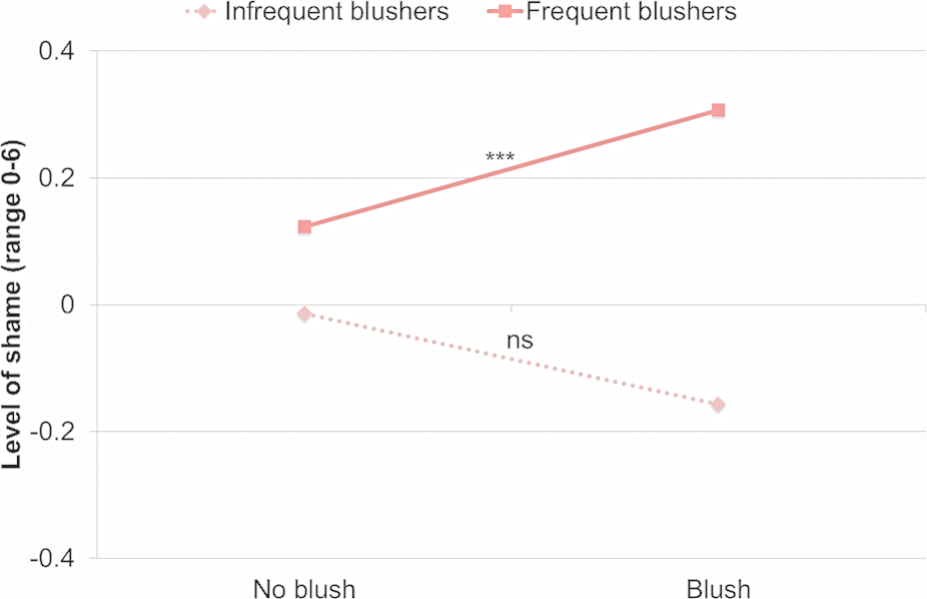
Shame during blushing and non-blushing events in more and less frequent blushers. *Note*. ***p < 0.0001; ns = not significant.

Shame was not significantly predicted by Blushing, *b* = 0.02, *F*(1,7006) = 0.14, *p* > 0.70, *d* = 0.01. Shame was significantly predicted by Blushing Frequency, *b* = 0.23, *F*(1,623) = 18.80, *p* < 0.0001, *d* = 0.35, and by the interaction, *F*(1,7040) = 8.43, *p* < 0.004. In infrequent blushers, blushing was not significantly associated with levels of shame, *b* = -0.14, *t*(7031) = 1.35, *p* > 0.17, *d* = 0.03. In frequent blushers, blushing was associated with higher levels of shame, *b* = 0.18, *t*(7014) = 5.56, *p* < 0.0001, *d* = 0.13. Frequent blushers, *M* = 0.12, *SE* = 0.12, reported more shame than infrequent blushers, *M* = -0.01, *SE* = 0.12, when they were not blushing, *t*(111) = 3.08, *p* < 0.003. Frequent blushers, *M* = 0.13, *SE* = 0.12, also reported more shame than infrequent blushers, *M* = -0.16, *SE* = 0.16, when they were blushing, *t*(3240) = 3.93, *p* < 0.0001. However, the difference in shame was larger when infrequent and frequent blushers blushed than when they did not blush, *t*(7040) = 2.90, *p* < 0.004. See Fig. 1 for an illustration.

The feeling of exposure was significantly predicted by Blushing, *b* = 0.24, *F*(1,6954) = 10.06, *p* < 0.002, *d* = 0.08, and Blushing Frequency, *b* = 0.06, *F*(1,260) = 6.36, *p* < 0.02, *d* = 0.31. The interaction term was not a significant predictor, *F*(1,6979) = 0.54, *p* > 0.46. Participants felt more exposed when they blushed, *M* = 0.39, *SE* = 0.18, than when they did not blush, *M* = 0.15, *SE* = 0.16. Frequent blushers felt more exposed, *M* = 0.44, *SE* = 0.17, than infrequent blushers, *M* = 0.10, *SE* = 0.19.

**Fig 2:**
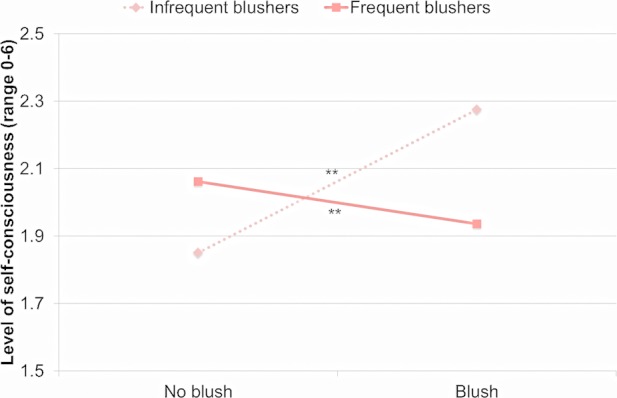
Self-consciousness during blushing and non-blushing events in more and less frequent blushers. *Note*. **p < 0.01.

Self-consciousness was not significantly predicted by Blushing, *b* = 0.15, *F*(1,6931) = 2.49, *p* > 0.11, *d* = 0.04, and by Blushing Frequency, *b* = -0.28, *F*(1,172) = 0.06, *p* > 0.80, *d* = 0.04. The Blushing by Blushing Frequency interaction was significant, *F*(1,6942) = 7.85, *p* < 0.006. This interaction is illustrated in Fig. 2. Blushing was associated with higher levels of self-consciousness in infrequent blushers, *b* = 0.43, *t*(6937) = 2.31, *p* < 0.03, *d* = 0.06, but with lower levels of self-consciousness in frequent blushers, *b* = -0.13, *t*(6931) = -2.18, *p* < 0.03, *d* = -0.05.

In summary, the state of blushing was positively associated with embarrassment or feelings of exposure in both frequent and infrequent blushers. Frequent blushers sometimes experienced higher levels of shame when they blushed (or lower levels of self-consciousness), whereas infrequent blushers sometimes experienced higher levels of self-consciousness when they blushed.

**Pleasant and unpleasant affect.** See Table 1 for F-statistics. Unpleasant affect was significantly predicted by Blushing, *b* = 0.17, *d* = 0.07, and by Blushing Frequency, *b* = 0.31, *d* = 0.62. The Blushing by Blushing Frequency interaction was not a significant predictor. Participants generally reported more unpleasant affect when they blushed, *M* = 0.67, *SE* = 0.14, than when they did not blush, *M* = 0.50, *SE* = 0.12., and frequent blushers generally reported more unpleasant affect during social encounters, *M* = 0.84, *SE* = 0.13, than infrequent blushers, *M* = 0.33, *SE* = 0.14.

**Fig 3:**
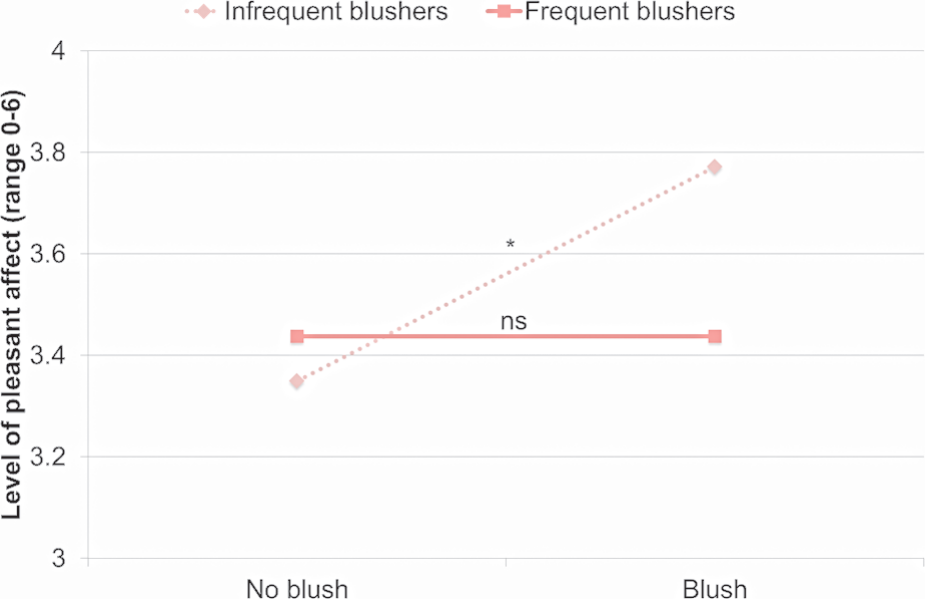
Pleasant affect during blushing and non-blushing events in more and less frequent blushers. *Note*. *p < 0.05; ns = not significant.

Pleasant affect was not significantly predicted by Blushing Frequency, *b* = -0.17, *d* = 0.09. Pleasant affect was significantly predicted by Blushing, *b* = 0.21, *d* = 0.05, and by the Blushing by Blushing Frequency interaction. See Fig. 3 for an illustration. For frequent blushers, pleasant affect was not associated with the state of blushing, *b* = 0.00, *t*(6941) = -0.01, *p* > 0.99, *d* = 0.00. In contrast, infrequent blushers experienced more pleasant affect when they were blushing than when they were not, *b* = 0.42, *t*(6957) = 2.26, *p* < 0.03, *d* = 0.05.

In sum, the state of blushing was positively associated with unpleasant affect in both frequent and infrequent blushers. Additionally, infrequent blushers sometimes experienced higher levels of pleasant affect when they blushed, whereas frequent blushers did not.

**Social behaviors**. See Table 1 for *F*-statistics. Levels of dominant behaviors, *b* = 0.63, *d* = 0.01, submissive behaviors, *b* = -0.31, *d* = 0.00, quarrelsome behaviors, *b* = -1.26, *d* = 0.01, and agreeable behaviors, *b* = 0.96, *d* = 0.01, were not significantly different during blushing events compared to non-blushing events.

Frequent and infrequent blushers did not differ significantly in terms of quarrelsome behaviors, *b* = 2.64, *d* = 0.09, and agreeable behaviors, *b* = -2.85, *d* = 0.09. However, frequent blushers generally reported fewer dominant behaviors, *M* = 6.15, *SE* = 3.84, than infrequent blushers, *M* = 11.07, *SE* = 4.21, *b* = -3.70, *d* = 0.12. Moreover, frequent blushers generally reported more submissive behaviors, *M* = -9.44, *SE* = 3.88, than infrequent blushers, *M* = -14.96, *SE* = 4.25, *b* = 3.93, *d* = 0.14. The interaction term was not a significant predictor of social behaviors.

In short, altered social behaviors, specifically dominance and submissiveness, were associated with trait blushing frequencies but not with the state of blushing.

**Participants’ perceptions of others.** See Table 1 for *F*-statistics. Perceptions of others’ power were not significantly different during blushing events compared to non-blushing events, *b* = 0.07, *d* = 0.01, nor were perceptions of affiliation, *b* = -0.15, *d* = 0.02. However, frequent blushers generally perceived interaction partners as more powerful, *M* = 6.95, *SE* = 0.34, than infrequent blushers, *M* = 6.42, *SE* = 0.39, *b* = 0.30, *d* = 0.17. Moreover, frequent blushers generally perceived interaction partners as less affiliative, *M* = 7.33, *SE* = 0.36, than infrequent blushers, *M* = 7.88, *SE* = 0.41, *b* = -0.30, *d* = 0.15. There were no significant state by trait interactions. Thus, altered perceptions of interaction partners were only associated with trait blushing frequencies.

**Social interaction length.** The length of social encounters was predicted by Blushing, *b* = 16.44, *F*(1,6889) = 14.63, *p* < 0.0002, *d* = 0.09. Encounters that involved blushing lasted significantly longer, *M* = 66.58 min, *SE* = 9.93, than encounters that did not involve blushing, *M* = 50.15, *SE* = 8.97. Blushing Frequency, *b* = -4.43, *F*(1,485) = 0.01, *p* > 0.93, *d* = 0.01. The Blushing by Blushing Frequency interaction, *F*(1,6947) = 3.51, *p* > 0.06, did not significantly predict social interaction length. As the results indicated that longer encounters may have provided more opportunities for blushing, we repeated all state-trait analyses with social interaction length as an additional covariate. This did not meaningfully change the results (data not shown).

### Associations between trait measures and ECR blushing frequency in Sample 2

Descriptive statistics, reliabilities, and zero-order correlations of the blushing-associated trait measures are displayed in Table 2. Two missing BFNE scores and four missing SIAS scores were imputed using the sample mean. Reliability was high for the BFNE, the BPS, and the SIAS; moderate for the BTSQ-Blushing; and modest for the FQ-Social. See Table 2 for the Cronbach coefficient alphas. Sample 2 women had higher BPS scores, *t*(62) = -3.54, *p* < 0.0009, FQ-Social scores, *t*(62) = -3.04, *p* < 0.004, and BFNE scores, *t*(62) = -3.48, *p* < 0.001, than Sample 2 men. There was no significant gender difference on the BTSQ-Blushing, *t*(41) = -1.90, *p* > 0.06, nor on the SIAS, *t*(62) = -1.47, *p* > 0.14.

**Table 2:** Means, standard deviations, reliabilities, and correlations on blushing-associated measures administered in Sample 2.

	M	SD	1	2	3	4	5	6	7	8
1. BPS^a^	35.39	8.74	(0.89)							
2. BTSQ-Blushing^a^	30.46	15.95	0.47***	(0.77)						
3. SIAS^a^	20.77	9.82	0.52***	0.59***	(0.88)					
4. FQ-Social^a^	10.80	4.73	0.27*	0.21	0.35**	(0.58)				
5. BFNE^a^	33.97	8.51	0.40**	0.38**	0.44***	0.25*	(0.90)			
6. Number of returned ECR forms	69.58	30.49	0.13	0.22	0.11	0.17	0.25*	(0.81)		
7. Social interaction length (min)	43.13	40.00	0.06	0.00	0.04	-0.02	0.08	-0.41***	(0.75)	
8. Blushing frequency^b^	7.42	8.67	0.54***	0.29*	0.38**	0.05	0.24	-0.10	0.15	(0.66)

*p < 0.05

**p < 0.01

***p < 0.001

^a^Assessed using trait measures.

^b^Assessed using ECR; correlations performed using log-transformed values.

Values in parentheses along the diagonal are Cronbach coefficient alpha’s indicating the extent of agreement among scale items. For the number of returned ECR forms, social interaction length, and blushing frequency the numbers represent the correlations between scores from week 1 versus week 2 of the study.

Blushing frequencies assessed using ECR were log-transformed to correct for their left-skewed distribution. BPS scores, BTSQ-Blushing scores, SIAS scores, and log blushing frequencies were inter-correlated (Table 2). Blushing frequency assessed using ECR was higher in participants with a higher self-reported tendency to blush often, with more fear of blushing, and with higher trait levels of anxiety during social interactions.

Log blushing frequency was not significantly correlated with FQ-Social scores. Moreover, when the FQ-Social and the BTSQ-Blushing were both entered as predictors of log blushing frequency in a multiple regression model, only the parameter estimate for the BTSQ-Blushing was significant, *b* = 0.02, *t*(1) = 2.38, *p* < 0.03. The parameter estimate for the FQ-social was not significant, *b* = 0.00, *t*(1) = -0.10, *p* > 0.91. Thus, blushing frequency assessed using ECR was more closely associated with a fear of blushing than with a tendency to avoid socially evaluative situations.

## Discussion

Blushing was found to occur on average about once or twice a day. There was substantial variability among individuals in the frequency of blushing. Some participants never blushed during the two weeks of the study while others blushed during almost half of their recorded social encounters.

Women were more likely to report blushing than men. While retrospective questionnaire studies have not consistently found a gender difference in blushing [4,31], laboratory studies have found that women who are embarrassed have greater increases in cheek temperature and are more likely to be perceived as blushing [6,28]. Thus, results support a gender difference in blushing both when evoked experimentally in the laboratory and when reported, without substantial retrospection, in daily life.

Consistent with previous studies in which blushing was studied retrospectively [31], using laboratory measures [28], or with literary examples [11,12], blushing was more common in interactions with individuals of the opposite sex and in interactions with a romantic partner. In contrast to Shields and colleagues [31] we did not find that blushing was more common when the interaction involved a partner with a higher status, i.e. a boss. However, few participants, who were university students, reported this type of interaction, so we may not have had enough statistical power to find the expected differences.

The state of blushing involved several intrapersonal correlates; participants felt more unpleasant, embarrassed, ashamed, and exposed when they blushed compared to when they did not blush. While this does not provide any information on the directionality of the observed associations (blushing might cause embarrassment or an embarrassing situation might cause someone to blush), these results are consistent with the idea that blushing is undesirable [1,21,54]. Importantly, the results are unlikely to be explained by differences in the blushing context in terms of participant gender, (primary) partner gender, whether the social encounter took place in a group, and location, since we statistically controlled for these situational factors in the analyses. This reduces the likelihood that blushing-associated embarrassment occurred during different *types of* social encounters than blushing-associated shame (though see the Limitations section below).

The absence of clear links between the state of blushing on the one hand and social behaviors and perceptions on the other hand was unexpected. However, it has been argued that the blush is often consciously ignored by both the blusher and the observer [41]. This may be because the blush serves as an indication that the blusher did not intend for an undesirable event, such as a mishap or transgression, to happen and is motivated to prevent it from happening again [30]. Thus, blushing may reduce the need for displaying the submissive and agreeable behaviors that would probably be necessary if a mishap or transgression were to occur and the actor did blush.

Independent of whether participants blushed or did not blush during a social encounter, frequent blushers reported lower levels of dominant behaviors and higher levels of submissive behaviors than infrequent blushers, and frequent blushers perceived others to be more powerful and less affiliative. Moreover, blushing frequency moderated the association between blushing and shame, between blushing and self-consciousness, and between blushing and pleasant affect. In participants who blushed frequently, blushing was associated with higher levels of shame than in participants who blushed infrequently. In addition, while levels of self-consciousness in frequent blushers were high even when they did not blush, infrequent blushers were self-conscious only when they blushed. Blushing was associated with higher levels of pleasant affect specifically in infrequent blushers. The results suggested that even though blushing is often linked to unwanted interpersonal situations, in some individuals blushing also occurred during positive social encounters.

### Blushing is undesirable

Consistent with previous findings that many people find blushing unpleasant [18,54,55], we found that our participants, who were young adults, generally experienced more negative feelings when they blushed than when they did not blush. Moreover, we found that different blushing events might be uniquely associated with feeling embarrassed, ashamed, or exposed. This provides further support for distinguishing between embarrassment and shame [56]. Shame typically involves stronger negative feelings and both more global and more enduring negative social cognitions while embarrassment typically involves more specific and transient situations. Thus, experiencing shame is more undesirable than experiencing embarrassment. In addition, blushing and shame are less likely to co-occur than blushing and embarrassment [57]. We found that levels of embarrassment were elevated during blushing in all participants, but levels of shame were only elevated during blushing among frequent blushers. The ECR methodology we employed does not allow for tests of causality. Nevertheless, we might speculate that the experience of embarrassment may trigger blushing in both frequent and infrequent blushers, but blushing may trigger shame only in frequent blushers.

Blushing when feeling ashamed is thought to serve an appeasement function [10,13,19,23]. However, frequent blushers may overestimate the impact of a blush [58] and over time develop negative beliefs about blushing [55]. They may come to believe that they should not blush and that blushing is a sign of weakness. People who consider frequent blushing a character flaw likely experience shame when they blush. Research using retrospective measures has found that frequent blushing is common among people with a fear of blushing [4] and among people with a fear of negative evaluation [5]. Similarly, in our study blushing frequency assessed in real-time was positively associated with fear of blushing. However, blushing frequency was not associated with a more general tendency to avoid socially evaluative situations, which is consistent with the idea that fear of blushing represents a specific form of social anxiety [59,60].

Since expressions of embarrassment and shame signal a reduction in status and a wish to appease [9,13,21,25,56,61], we expected blushing to be associated with lower levels of dominant and quarrelsome behaviors and higher levels of submissive and agreeable behaviors. We also expected blushing to be associated with perceiving others as powerful and low in affiliation. However, there were no blushing-associated alterations in any of the behavioral and perceptual variables. Rather, altered behaviors and perceptions in terms of dominance-submissiveness and quarrelsomeness-agreeableness were more closely linked to the trait of blushing. Moreover, there were no state-trait interactions. It appears that between-person differences in social behaviors and perceptions reflect stable characteristics of young adults that do not increase in the event of blushing. The incidence of blushing phobia is higher among teenagers [59] than among young adults and the prevalence of blushing phobia decreases in the late teens [62]. It is possible that blushing may be more strongly linked to altered social behaviors and perceptions among teenagers than among young adults.

In sum, we found evidence that blushing is associated with feeling embarrassed, exposed, self-conscious and, in frequent blushers, shame. On the basis of these findings, we speculate that experiencing shame after blushing may increase fear of blushing, thereby increasing the likelihood of developing social anxiety disorder. Frequent blushing may help maintain fear of blushing, particularly since frequent blushers generally behave in a non-dominant and submissive way and perceive others as high in power or status and low in affiliation.

### Blushing may have positive effects

In spite of the previously described findings, several observations suggest that displaying a blush may not always be detrimental. First, while frequent blushers reported higher levels of shame when they blushed than when they did not blush, this was not found among infrequent blushers. Second, infrequent blushers reported more pleasant affect when they blushed than when they were not blushing. This was not found among frequent blushers. Third, we found no evidence for altered social behavior in the immediate context of blushing.

From these findings we might infer that occasional blushing may often be socially acceptable and might sometimes even be socially rewarding, such as when it has a remedial value following a mishap or transgression [10]. Moreover, as the blush often serves to communicate that the actor did not intend for a mishap or transgression to happen and is motivated to prevent it from happening again in the future [30], our finding support the idea that blushing may reduce the need for displaying the submissive and agreeable behaviors that would be necessary if the embarrassing or shameful event were to occur and the actor did not blush [9,19]. Particularly for people who blush infrequently, blushing might constitute an accurate sign of embarrassment or shame and thereby have a salutary effect on repairing social relationships.

### Limitations of our research

In the present study we employed an extensively validated ECR method [34,39,40,63]. ECR for the study of blushing has advantages over previously used methods but also some disadvantages. First, we asked participants to use structured forms to record aspects of their social encounters soon after they occurred without requiring qualitative descriptions of their content. We do not have detailed information about specific events, such as whether people started blushing because they felt embarrassed or ashamed or whether people became embarrassed or ashamed after they felt they were blushing. In other words, it is unclear exactly when blushing occurred during events that involved blushing. Indeed, due to the correlational nature of the ECR methodology, it is important to keep in mind that our findings cannot be interpreted in terms of cause and effect. ECR studies are best seen as complementary to laboratory studies in which blushing can sometimes be elicited.

A second limitation is that a variety of event-level variables that are known to influence the likelihood of blushing, such as being the center of attention or being involved in a self-presentational predicament, were not assessed. From our data analyses we excluded social encounters that involved phone conversations or the use of alcohol and we statistically controlled for several situational factors, but there may be other known and unknown factors that could have influenced our results, such as temperature [54] and medication use [64]. In particular, the reasons for blushing might have varied both between and within participants.

To maintain manageable demands on participants’ time, participants were instructed to record social encounters that lasted at least five minutes. Hence we missed shorter events, which might produce misleading findings if the unpleasant affect associated with blushing motivates people to engage in shorter interactions with others. However, in Sample 2 social interaction length was not significantly associated with blushing-associated trait measures or with blushing frequency assessed using ECR (Table 2), and in the combined sample blushing events were not briefer than non-blushing events.

A final limitation of our study is that blushing was measured subjectively using self-reports. A more objective measure may be facial blood flow which has been assessed in the laboratory [28,60,64,65] but this procedure is difficult to conduct in daily life. We relied on participants to report their own blushing. Participants may have been blushing but not reported it, because they did not realize they were blushing. We consider this unlikely because blushing happens suddenly and is intense. Nevertheless, observer ratings of blushing are necessary to exclude this possibility. Further, observers would be able to rate the reddening of the face and the various motoric signals often associated with blushing.

### Studying the blush: Opportunities for the future

Advantages of ECR for the study of blushing include assessment in daily life, good temporal stability, and the possibility to look at a range of related social behaviors. This opens up interesting possibilities for future studies. For example, one next step would be to compare individuals with a diagnosed blushing phobia with controls in terms of how they behave, feel, and perceive others during interactions with others.

More generally, the ECR method we employed could be adapted further. Additional changes in the social interaction record forms might help provide more insights into when and why people blush. For example, we could ask participants about the occurrence of mishaps and transgressions during their interactions with others, or we could ask them whether they did something others might not like, whether they felt others might be judging them, or whether they feared something would be disclosed, all of which are alleged elicitors of the blush. Further, while participants in the present study simply indicated whether they blushed or not, participants in future studies could be asked to rate the perceived intensity of their blush. Individuals with blushing phobia may report blushing at higher intensities than controls [54].

Participants might also be asked whether they observed blushing in their interaction partners. They may behave differently when others blush than when they do not blush. For example, if others appear embarrassed after a mishap, this may convey awareness of or concern about the impressions they make [66]. Thus, seeing someone blush may cause people to be more agreeable towards this person.

Observers can usually perceive the presence of a blush but have difficulty assessing its intensity. Studying dyads would permit asking both partners about their experience and about the observed blushing intensity. Individuals with a fear of blushing may overestimate the impact of their blush in some contexts more than in others [67].

Finally, in general there have been few studies on the experience of embarrassment, shame, and exposure in daily life. O'Grady and colleagues conducted a one-month diary study on alcohol use after embarrassing events [68]. Thomaes and colleagues conducted a two-week diary study on anger expressions after shameful events [69]. Participants were explicitly asked to report embarrassing or shameful events, which may have introduced a reporting bias. Future studies could assess the impact of embarrassment or shame on behavior by employing ECR to sample social encounters regardless of whether participants experienced embarrassment or shame during those encounters.

### Summary and conclusion

We found blushing in young adults to be positively associated with unpleasant affect and with other undesirable states such as embarrassment. Importantly, frequent blushers but not infrequent blushers showed a positive association between blushing and shame, whereas infrequent blushers but not frequent blushers showed a positive association between blushing and pleasant affect. Frequent blushers generally behaved less dominantly and more submissively, and perceived others in a more negative light. The present pattern of findings is consistent with the view that while blushing often may be maladaptive, for some people blushing may have positive effects, such as feeling more pleasant and perceiving others more positively.

This is the first study to report on the feelings, behaviors, and perceptions associated with blushing in daily life. In spite of some limitations, the ECR approach used in this study opens up several opportunities for further exploring the correlates of facial blushing during every-day social encounters.
