# Correction: Intrapersonal and Interpersonal Concomitants of Facial Blushing during Everyday Social Encounters

**DOI:** 10.1371/journal.pone.0128726

**Published:** 2015-05-26

**Authors:** The PLOS ONE Staff

## Notice of Republication

This article was republished on April 2, 2015, to correct an error in Dr. Marije aan het Rot’s name. This error affected how the article was indexed in PubMed, as well as the citation and the copyright statement. The publisher apologizes for the errors. Please download this article again to view the correct version.
